# Shattering Silence, Inviting Dialogue: Anti-Oppressive Occupational Therapy During the Genocide of Palestinians

**DOI:** 10.1177/00084174251356348

**Published:** 2025-07-09

**Authors:** Hiba Zafran, Brenda L. Beagan, Dominique Shephard, Heidi Lauckner, Karen Whalley Hammell, Katie Lee Bunting, Marie-Lyne Grenier, Pier-Luc Turcotte, Sara Abdo, Tal Jarus

**Keywords:** Human rights, Occupational justice/injustice, Practice guidelines, Professional ethics, Solidarity, Droit de la personne, éthique professionelle, justice/injustice occupationnelle, lignes directrices, solidarité

## Abstract

**Background.** Occupational therapists are obligated to promote human rights and are required to advocate for the profession's statements concerning social justice to align with its actions. **Purpose.** This Commentary provides an anti-oppressive perspective, developed from the different positions and identities of authors currently living in Canada, contending that the practices of occupational therapists cannot be viewed as disconnected from global conflicts such as the genocide of Palestinians, and providing guidance for those seeking to align their actions with the profession's espoused values and obligations. **Key issues.** Identifying anti-oppression as an ethical, moral, and professional imperative, this commentary articulates a principled examination of a complexified issue; providing suggestions for how occupational therapists, as individuals and as a profession, can engage in anti-oppressive practices through: (a) commitment to learning, (b) reflexive and reflective personal work, (c) the use of guiding frameworks, (d) building community, and (e) compassionate actions. **Implications.** Noting that there is never a neutral or apolitical position in the face of injustice, the paper invites dialogue, and provides suggestions and guidance for occupational therapists seeking to align their actions with their professional obligations in supporting human and occupational rights locally and globally.

## Résumé

**Description.** Les ergothérapeutes ont l’obligation de promouvoir les droits de la personne et de plaider pour que les déclarations de la profession concernant la justice sociale soient en phase avec ses actions. **But.** Ce commentaire offre une perspective anti-oppressive, développée à partir des différentes positions et identités de ses autrices et auteurs, qui vivent actuellement au Canada. Il soutient que les pratiques des ergothérapeutes ne peuvent être considérées comme étant déconnectées des conflits mondiaux tels que le génocide des Palestiniens, et fournit des orientations à ceux et celles qui cherchent à ce que leurs actions soient en phase avec les valeurs et les obligations de la profession. **Questions clés.** Identifiant l’anti-oppression comme un impératif éthique, moral et professionnel, ce commentaire se penche sur une question complexefiée, dont il articule un examen fondé sur des principes. Il fournit des suggestions sur la façon dont les ergothérapeutes en tant qu’individus et l’ergothérapie en tant que profession peuvent s’engager dans des pratiques anti-oppressives par : 1) l’engagement à apprendre; 2) le travail personnel réflexif et réfléchi; 3) l’utilisation de cadres de référence; 4) le renforcement de la communauté; et 5) la compassion. **Implications.** Notant qu’il n’y a jamais de position neutre ou apolitique face à l’injustice, cet article invite au dialogue, et fournit des suggestions et des conseils aux ergothérapeutes qui cherchent à aligner leurs actions sur leurs obligations professionnelles en soutenant les droits de la personne et les droits occupationnels à l’échelle locale et mondiale.

## Introduction

According to the World Federation of Occupational Therapists (WFOT, 2020) “WFOT, its Member Organizations, occupational therapists, assistants, and students are obligated to support occupational justice and human rights” (p. 2); thus, human rights advocacy is named as a core purpose of occupational therapy education and practice (WFOT, 2016). The *Competencies for occupational therapists in Canada* (ACOTRO et al., 2021) require occupational therapists, educational programs, students, and professional associations to “promote anti-racist, anti-ableist, and anti-oppressive practices” (p. 6), take “action to challenge oppression” (p. 19), and “advocate for an alignment between occupational therapy standards … and social justice” (F4.2), with particular attention to social position, contextual factors, privilege, and power. The Canadian Joint Position Statement *Toward Equity and Justice: Enacting an Intersectional Approach to Social Accountability in Occupational Therapy* (CAOT et al., 2024) also identifies the responsibility for occupational therapists in Canada to use the opportunity afforded by their “position, experience, and privilege … to advance equity and social justice” (p. 3), as well as “influence the profession and its contribution to society” (ACOTRO et al., 2021, F4.4).

Our aim in this commentary is to urge conversation and action within the profession about our role in responding to global conflicts currently taking place, with a particular focus on the genocide of Palestinians. Because of the responsibilities previously outlined, our practices as occupational therapists cannot be viewed as disconnected from global conflicts such as the genocides of Indigenous peoples across Turtle Island, people in the Darfur region of Sudan, the Rohingya People in Myanmar, Armenian People in Azerbaijan, the Hazara People in Afghanistan, Uyghur Muslims in China, and other devastating conflicts such as the Russian invasion of Ukraine. Here, we focus on the oppressive silence surrounding the genocide of Palestinians, specifically because discussions and actions have been pointedly avoided, or actively and punitively silenced, while genocidal actions are actively supported by Canadian policies and funds (e.g., McGregor, 2024). It is not that one conflict matters more than another, but rather that the institutional, public, and professional (non)responses to news about and solidarity against the genocide of Palestinians are retaliatory and complicit in light of clear evidence of ongoing crimes against humanity. The genocide of Palestinians constitutes a current global crisis that illustrates that our profession does not consistently act in accordance with its espoused values and responsibilities, pointing to the need for strategies to better enact anti-oppressive practices in the face of human rights violations.

We begin by drawing attention to a call for international solidarity from our Palestinian colleagues in Gaza that has remained unanswered by our profession's international and national bodies. Then we outline why anti-oppressive practice is an ethical, moral, and professional imperative for occupational therapy in this context. We address questions about the genocide of Palestinians posed by students, educators, and occupational therapists, and invite readers’ reflective engagement. Finally, we explore what individual occupational therapists, as well as our profession, can do as we bear witness to this genocide, knowing that these reflections and actions will have relevance to other genocides and global conflicts now and in the future.

### A Call for Solidarity Remains Unanswered

On October 27, 2024—World Occupational Therapy Day—a Palestinian occupational therapist in Gaza sent an Instagram message to the international occupational therapy community, describing their appalling situation in Gaza, amid “rubble, blood, body parts, starvation and displacement,” and saying “we are dying here…You must say something if only to let us know you still remember us here, **where is the WFOT?**” (El Hallaq, 2024, emphasis in original).

Although the Canadian Association of Occupational Therapists (CAOT) was well-positioned to eschew the bystander effect and act in accordance with its own principles (e.g., “No silence in the face of inequality and injustice,” CAOT, 2020,  n.d.), CAOT has sent just one message to its constituent members about the unnamed genocide in Palestine, via *OT Weekly*, on October 26, 2023, naming it a “time of difficulty”; this was not updated as the genocide unfolded despite multiple petitions from members and international third-party investigative reports. The systematic obliteration of the Gazan healthcare system (Abi-Rached & Reinhart, 2024; Manduca et al., 2024) in which our occupational therapy colleagues worked violates International Law (Homer, 2024) and constitutes a war crime (Faddoul et al., 2024). “Educide” and the targeted bombing of every Gazan university (Gordon & Turner, 2024)—including Al Aqsa University, where our occupational therapy academic colleagues taught and researched (Babish et al., 2024), and the imperilment of the work of the Palestinian Occupational Therapy Association: all have been (and still are) met by silence from the leading organizations in our profession. Clearly, these human rights violations impact the ability to engage in daily occupations “without risk to safety” (WFOT, 2019). CAOT's silence has been heard very clearly in Gaza: “@caotace You are complicit in the genocide of Palestinians. We will never forget. We will never forgive” (El Hallaq, 2024).

On the same day that our Palestinian colleague called on our profession to “say something,” an anonymous collective of 60 Canadian occupational therapists, students, and faculty members published a peer-reviewed paper entitled “*Occupational therapists speaking for justice and human rights: From complicit silencing to collective resistance”* (AnonymOT Collective, 2024). The paper was brought to the attention of the CAOT's membership via *OT Weekly* on November 21, 2024 (CAOT, 2024), as one of a series of “articles of interest,” providing a content warning that the paper contained “depictions of violence” and referred to the “genocide in Gaza.” The AnonymOT Collective documented historical and current health, social and occupational injustices in the occupied Palestinian territories and condemned the silencing of occupational therapists and students in Canada who have been “erased, denied, and harmed” for speaking against this genocide (AnonymOT Collective, 2024, p. 15). The paper concluded that our professional organizations have not enacted their own professional obligations and ethical standards for human rights’ advocacy and have failed to “stand against the production of disability on an industrial scale” (AnonymOT Collective, 2024, p. 21).

The AnonymOT Collective raised issues that we have also observed and experienced: students have been required by their programs to remove anti-genocide social media posts that cited the Canadian competencies under threat of withdrawal from fieldwork placements, faculty who name and honor the murdered Palestinian and Israeli occupational therapists are sanctioned with imposed changes to their teaching responsibilities, clinicians who formally report Islamophobic discrimination against clients in the workplace are instead investigated themselves by their union for the “antisemitic” submission of such a report, occupational therapists wearing cultural symbols of their homeland(s) are deemed “hostile,” and more. The silence/silencing has increasingly polarized our professional settings and has further traumatized communities. Occupational therapy students have raised serious concerns with us about the presumed “neutral” stance of their professional bodies and academic programs, questioning whether the profession's ethical compass aligns with its stated commitment to uphold occupational and human rights (CAOT, 2020; CAOT et al., 2024; WFOT et al., 2014, 2016, 2019, 2020).

Examining what contributes to the profession's silence may help us to better live up to our espoused values in this current genocide and future global events. While silence can provide space for internal processing and sense-making, and can protect against anticipated punitive responses, in this paper we explicitly focus on the silence that signals avoidance, discomfort, refusal, or disapproval and that supports harm; the kind of silence that viscerally feels like a door slammed shut (Ahmed, 2019), an intentional and systemic erasure of the issue and the questions it raises. This kind of silence contributes to dehumanization (Alyan, 2023), casting the “other” as lesser, diminishing the humanity of all involved (Fanon, 1952), and foreclosing painful but necessary transformational dialogues.

### Anti-Oppression as an Ethical, Moral, and Professional Imperative

As we were each witnessing this intolerable and unethical silence with distress, we reached out to each other as trusted colleagues, thereafter forming a close-knit web: a supportive group committed to learning from each other, drawing from the strengths of our diversity (Hammell, 2019), and standing together in solidarity to shatter the silence. We are a group of Canadian academics striving to live up to our *responsibility* to use the opportunity afforded by our “position, experience, and privilege … to advance equity and social justice” (CAOT et al., 2024, p. 3). We are racialized and white; we are from different class backgrounds, ethnicities, migration histories, and professional profiles; we embody a diversity of genders and sexualities; we are enabled and disabled; we are young, we are old; we have diverse religious backgrounds, and no religious identification. We are partnered and single, parents and aunts/uncles. Some of us have no direct experience of victimization by genocide, and some have close and extended family members, friends, communities, and ancestors directly impacted by the intergenerational trauma and slaughter of peoples on their own land—including Palestinians. By describing ourselves in this way, we acknowledge the importance of our social identities while prioritizing privacy over both tick-box, reflexive performance (Sibbald et al., 2025), and identity politics (Jabr, 2025). What matters more than our positionality statement, and the assumptions it may elicit (who we are), is the intentional “repositioning” of ourselves against genocide and structural violence (what we stand for) (Byrne et al., 2020, p. 49).

This paper does not aim to offer a final word on world issues with lengthy or contested histories, but rather an opening for further conversation, reflection, and action. Though we approach these conversations from different social positions and identities, five principles unite us: (a) *solidarity* in opposing all forms of oppression and dehumanization; (b) ongoing attempts to *openly participate* in difficult conversations; (c) *deep (un)learning* of colonial and other systemic power asymmetries that structure occupational inequity and injustice, and that demand transformational work; (d) *holding ourselves accountable* to our profession's stated commitments to justice; and (e) that *addressing structural inequalities* and challenging supremacist discourses is necessary for the liberation of all. Speaking to our moral commitments, these ethics are all rooted in principles of anti-oppressive practice (ACOTRO et al., 2021) and commitments to anti-oppressive knowledge-in-action required by Section C of the Canadian competencies related to culture, equity, and justice (2021, pp. 13–14).

As occupational therapy educators and practitioners, we unreservedly condemn all forms of racism and support the need to confront the ways they—and other intersecting forms of oppression—weave through occupational therapy's practices, research, and education (Beagan et al., 2023; Beagan et al., 2022; Désormeaux-Moreau et al., 2024; Grenier, 2020; Lavalley & Johnson, 2022; Turcotte & Holmes, 2023). Acknowledging the genocide being conducted by the state of Israel against the Palestinians does not reduce, erase, or lessen the need to address antisemitism and other forms of discrimination. The loss of any life to conditions of injustice is deplorable. We grieve the Israelis who were killed or taken hostage on October 7, 2023, and the innumerable Palestinians and Lebanese^1^ who—in their own words—have been martyred in the genocide. The rise of anti-Jewish, anti-Muslim, and anti-Arab racism and hate crimes across Canada is not acceptable. The suffering of families and communities living at the intersections of Arab, Muslim, Syrian, Christian, Palestinian, Druze, Lebanese, Jewish and Israeli identities^2^ should not be in competition with each other. We resist the supremacist hierarchy of the worthiness of lives based on social identities. Instead, we enact an anti-oppressive lens, which focuses on historically rooted structural power dynamics and discourses that justify and enact the significant erasure, marginalization, and deaths of some groups to serve the interests of another (Pooley & Beagan, 2021). This frames our focus on Palestinian human rights abuses (UN General Assembly, 2024), which include ethnic cleansing, apartheid, and genocide (Pappe, 2024).

The United Nations contends that “The [International] Court's findings should be widely disseminated to ensure that the illegality of Israel's presence in the occupied territory is fully understood at all levels of the government and reflected in public documents and education systems” (United Nations’ Office of the High Commissioner for Human Rights, July 30, 2024). Such a structural perspective recognizes that the damage experienced across all groups living in the occupied Palestinian territories as well as the modern state of Israel occurs within an unjust overarching system (Bakan & Abu-Laban, 2024). The century-long enforced power asymmetry, contested and continually shifting borders, and system of apartheid (Federman, 2024; Pappe, 2024; Roth, 2025) is upheld by intolerance for/silencing of expressions of support for the rights of Palestinians, including state repression of Israelis who dissent with apartheid and unlawful occupation, and/or refuse to participate in the required military term, and genocide (Academia for Equality, 2025; Angel, 2023; Refuser.org, 2025; Rosemen, 2023; Rosenfeld, 2023; Voices Against War, n.d.; Ziv, 2024). This silencing is proposed as one feature of Anti-Palestinian Racism (Canadians for Justice and Peace in the Middle East, 2022; Rimawi et al., 2024), a distinct (and currently deadly) form of racism declared a priority by the Government of Canada (2024). Actionable dialogue must shatter this silence.

### Questions Posed by Occupational Therapists and Students

When they feel safe to ask us, students and colleagues have posed questions about the ongoing conflict in Palestine and Israel that warrant thoughtful responses and lay the foundations for ongoing, open dialogue, and critical reflexivity. Here, we openly state and engage with several of these questions.

### Is It Permissible to Name “Genocide” in Gaza?

The term *genocide* was coined and defined by Polish-Jewish lawyer Raphael Lemkin in 1942 as a foundation for the 1948 UN Convention on the Prevention and Punishment of the Crime of Genocide (Irvin-Erickson, 2017). Genocide is a crime committed with “the intent to destroy a national, ethnic, racial or religious group, in whole or in part,” by killing, causing serious bodily or mental injury, or deliberately inflicting destructive life conditions (UN, 1948). It is argued that Lemkin's intention was both moral and legal; that is, the term serves both as a legal framework for crimes against humanity, as well as providing a moral language for the witnessing and experiencing of such crimes (Kinstler, 2024). Morally, it is therefore important to witness and name that Palestinians have endured, and continue to experience genocide (Docker, 2012; Lendman, 2010; Nijim, 2023; Pappe, 2024; Robinson, 2024). The violence of the 1948 Nakba (“catastrophe” in Arabic) in historical Palestine that resulted in the displacement of three-quarters of a million Palestinians, transgenerational refugee status, and innumerable massacres is now being proposed as a legal concept to name the ongoing structural oppression that includes genocide (Eghbairah, 2024).

In late October 2023, Palestinian human rights organizations, Jewish civil society groups, Holocaust and genocide studies scholars, and a collective of more than 800 practitioners and scholars of international law were already warning of an escalation of the genocide in Gaza (Third World Approaches to International Law Review, 2023). Within the month, an Israeli security cabinet member was referring to the “Gaza Nakba” and “Nakba 2023” (Tov, 2023), intentionally linking current events to the ongoing project of occupying Palestine, including through ethnic cleansing (Pappe, 2007, 2024). By the end of 2023, Gaza was described as a “graveyard for children” (Elder, 2023), with the death toll of civilians, journalists, and an estimated 1,000 healthcare workers being proportionally higher than any other war in the last century (Acosta, 2024; Oxfam, 2024; Salam, 2024; United Nations’ Office of the High Commissioner for Human Rights, 24 September, 2024). In January 2024, the International Court of Justice deemed the situation a “plausible genocide” (International Court of Justice, 26 January, 2024).

At the time of writing this paper thousands of children and adults have sustained life-altering injuries and compounded intergenerational trauma because of indiscriminate bombing (Faddoul et al., 2024), targeted attacks (Abu Saif, 2024), deliberate starvation (King et al., 2025) and hypothermia (UN News, 2025). Gazans are prevented from accessing medical and surgical treatment for cancer and other life-threatening conditions (Coghlan et al., 2024; Faddoul et al., 2024; Homer, 2024; Manduca et al., 2024), with caesareans, amputations, and other surgeries being undertaken without adequate—or any—anesthesia (Mohd Fathil & Shariffuddin, 2024). There are now multiple investigative reports outlining evidence for a genocide from legal, humanitarian, and healthcare perspectives (Albanese, 2024; Amnesty International, 2024a, 2024b; Baltman & Goldberg, 2025; Mofokeng, 2023; Sultany, 2024). The hesitation of our profession to acknowledge these human rights violations—violations that clearly align with acts of genocide—warrants deep, critical self-reflection. Perhaps a more suitable question for our profession and ourselves would be, is it permissible *not* to name what is happening in Gaza a genocide?

### If I Critique the Government of Israel or Say I Am Against the Genocide of Palestinians, Is This Antisemitic?

Israel is a state populated not solely by Jews, but by Muslims, Christians, Druze, Bahá’i, and others of diverse ethnic origins and religious traditions (Pew Research Center, 2016). Criticizing Israeli state policies and actions is neither antisemitic nor anti-Jewish (CAUT; Canadian Association of University Teachers, 2020; IJV; Independent Jewish Voices Canada, 2024; Roth, 2025). There are diverse perspectives on the relationship between Jewish peoples and a nation of Israel; it is beyond the scope of this paper to critically examine the competing and multifold meanings, framings, and effects of Zionism (Abdo, 2024; Pappe, 2017, 2024; Salamanca et al., 2012). Regardless, naming the crimes against humanity unfolding in Palestine is not antisemitic, just as criticizing the violations committed by Hamas is not anti-Palestinian (International Criminal Court, 2024). We ought to be able to critically debate the legality of armed resistance under unlawful occupation (United Nations, 1990), regret and lament the impacts of such violence, and simultaneously stand against genocide. Criticizing the actions of a government, military, or a political party is neither racism nor discrimination against a particular ethnic identity or religion. Rather, it is the right and responsibility of global citizens to critique the actions of any government—including our own Canadian government (Middle East Monitor, 2025)—when policies and practices lead to crimes and injustice.

The traumatic weaponization of the term “antisemitic” (Pappe, 2024) to deflect or silence critiques of Israeli political and military practices has proven remarkably effective (Klein, 2025; Roth, 2025). It is helpful to be clear-thinking about the distinction between a state/government and an ethnic/religious group; for example, critiquing imperialist policies implemented by the British government is not anti-Christian. Antisemitism, or anti-Jewish racism, remains prevalent and unacceptable, and should be named and opposed. But opposing state-mandated genocide is not antisemitism (Chomsky & Pappé, 2015).

### Why Is There Such Hesitation and Inaction Within Occupational Therapy Around Such an Important Topic?

Beyond instances of overt censorship from our professional organizations and institutions, at an interpersonal level, there are multiple reasons for the silence. The intergenerational traumas of Palestinian, Lebanese, Jewish, and Israeli peoples in Canada—even if the sources of trauma are different—complicate responses to the current conflict. The guilt the world feels about the Nazi Holocaust has led to a fear of being perceived as antisemitic, even within Jewish communities. Palestinian peoples have been silenced in various ways for decades, their own histories overwritten and erased. For descendants and survivors of other traumas and genocides, the reverberations may be overwhelming, provoking automatic survival responses that may include shutting down (“freezing”) or avoidance (Menakem, 2017).

For some in the profession, the hesitation derives from a lack of knowledge concerning the history of occupation in Palestine and the creation of the modern state of Israel (Khalidi, 2020), leading to anxiety about saying something offensive or uneducated. This is complicated by the long-standing anti-Palestinian bias in Western media that many of us rely upon to make sense of the world's situation (Hamad & Najm, 2024; Khazzal, 2024). The challenge of being misinterpreted as racist or against specific religious groups (e.g., the perception that speaking out against the genocide of Palestinians means you do not care about anti-Jewish discrimination) can lead to avoidance. For many, it is easier to simply be a bystander, striving for a position of feigned neutrality. This stance is inevitably not neutral, when the pain of one group is foregrounded in mainstream discourses and supported by economic powers, while the truths of another group with less power are actively suppressed.

### Why Shouldn’t Occupational Therapists Remain “Neutral” or Silent?

The notion of “medical neutrality” does not entail remaining “neutral” or silent in the face of injustice. As indicated by the AnonymOT Collective (2024), this principle was developed in the context of violent conflicts “to ensure that healthcare is delivered to all wounded people and that all parties refrain from attacking healthcare facilities” (p. 20). There is never a neutral or apolitical position in the face of injustice. To stay silent is to align with the oppressors; silence signals approval of, and supports the status quo of current power dynamics—in this instance, genocide. It is no coincidence that South Africa was the first state to place a formal accusation of genocide against Israel at the International Court of Justice, enacting Archbishop Desmond Tutu's well-known statement:

If you are neutral in situations of injustice, you have chosen the side of the oppressor. If an elephant has its foot on the tail of a mouse, and you say that you are neutral, the mouse will not appreciate your neutrality. (cited in McAfee Brown, 1984, p. 19)

As reminded by the AnonymOT Collective (2024), WFOT has articulated the obligations and responsibilities of occupational therapists in promoting human rights through a series of Position Statements (WFOT, 2006, 2016, 2019, 2020, 2024). WFOT has declared, for example, that “the profession needs to speak out about the occupational implications of policy when it affects health, rights, and peace” (2014, p. 3), and has issued “a call to action … to support the global movement for justice, advocate for human rights, and lead change” (2020, p. 1). Nowhere does the WFOT identify the option for occupational therapists to be silent or “neutral” in the face of injustice or human rights abuses.

Canadian occupational therapy competencies include the requirement for students and practitioners to respond to, and to speak out if they witness abusive, unethical, or oppressive behavior (ACOTRO et al., 2021; E1.8). We take an oath to do no harm, a bioethical principle developed in the wake of the Nazi Holocaust (Chelouche, 2021). Indeed, as health care professionals “our ethical obligation is to speak out, as much as possible” (Fernandes, 2024, p. 13) and use the position of privilege afforded to us to support human rights (United Nations’ Office of the High Commissioner for Human Rights, n.d.). When oppression, violence, and atrocity are normalized—when they become the dominant ways of doing and being—saying and doing nothing in opposition (in a pretense of remaining “neutral”) becomes complicity. As Israeli psychologist Nissim Avissar argues,

While aspiring to change and well-being, therapists cannot just stand aloof and passively (and most unwittingly) take part in the continuation of hardship and distress. Remaining silent, ignorant or passive would make us accomplices to the production of human suffering, and would constitute a betrayal of our basic values as therapists. (2008, p. 170)

Our silence, our feigned “neutrality” in the context of grave social injustices simultaneously tells others—our colleagues, students, friends, service-users—that we cannot be counted on to stand up to oppression. Indeed, because our silence harms, it is itself an act of oppression.

### How *Should* Health-Care Professions Globally Respond to Genocide?

The UN's special rapporteur on the occupied Palestinian Territories has declared that “[t]he entire population of Gaza is at risk of dying in a genocide that has been announced and executed under our watch” (Albanese, cited in Mahase, 2024, p. 1). Disabled people—especially wheelchair users and those with visual impairments—have been unable to flee from relentless bombing (Burke & Tantesh, 2024; United Nations’ Office of the High Commissioner for Human Rights, 27 May, 2024), and individuals with intellectual and other disabilities have been killed (Keane, 2024). Physicians have proclaimed the “moral imperative and professional duty” (Ijaz & Habib, 2024, p. e1) to uphold their profession's ethical principles and espoused commitment to addressing the causes of ill health and human suffering in Gaza (Abbas & Mitchell, 2024; Coghlan et al., 2024; Dehghan et al., 2024; Khan & Tinua, 2024), and have identified just two choices available to healthcare professionals: “resistance or collaboration” (Mohd Fathil & Shariffuddin, 2024, p. 8). Choosing “resistance,” the Canadian Association of Midwives (2023), for example, spoke out promptly to uphold the rights of pregnant women and infants in Gaza. The occupational therapy profession in Canada made a different choice: remaining silent/enforcing silence.

Healthcare professionals are being called on as “defenders of human rights” that impact health and well-being (United Nations’ Office of the High Commissioner for Human Rights, n.d.). An evidence-based definition of medical genocide is being proposed by the United Nations, both to stop the repression of healthcare workers, and to use healthcare data to provide an early index of genocide that will enable prevention and accountability to be prioritized (Abughnaim et al., forthcoming). This aligns with the United Nations’ Convention on the Rights of Persons with Disabilities, “in particular during armed conflicts and foreign occupation” (United Nations Office of the High Commissioner, 2006, section u). Killing and disabling, regardless of the context, demand opposition by all health professions—including ours.

The international occupational therapy profession does not have a proud history of human rights’ advocacy, as evidenced by its literature. In 1997, for example, the South African Association of Occupational Therapists admitted its passive complicity with apartheid policies that denied millions of South Africans their human and occupational rights (Watson, 2008). It was encouraging that when Black Lives Matter protests erupted following the killing of George Floyd by police officers in the USA, CAOT was among the occupational therapy associations that issued public statements. Declaring “No silence in the face of inequality and injustice,” and identifying the imperative “to remind ourselves of who we are and what we stand for … and be authentic in aligning human rights with our everyday actions, in pursuit of a world that is fair, just, caring and compassionate,” we were assured: “CAOT will not be silent” (CAOT, 2020). And, again, in 2022, CAOT claimed to be standing “in solidarity with Ukrainian people as they defend themselves against aggression,” claiming to be “distressed and disheartened by the atrocities being committed in Ukraine and around the world by authoritarian regimes” (CAOT, 2022). This solidarity was apparently contingent, and not a stance embraced for all those seeking to defend themselves against authoritarian regimes and illegal occupation.

The literatures of other health-care professions exhibit a more robust history of aligning advocacy and action with espoused ethics. Thus, for example, during the 1940s, the medical literature featured papers documenting atrocities and denouncing fascism (Ephron, 1941); both the medical (Swiss & Giller, 1993) and nursing (Brandt, 2007) literatures have described efforts to label rape a war crime; with the literatures of medical ethics (Dowdall, 1991) and medicine (Nightingale et al., 1990) recording state-sponsored torture and institutionalized violence in South Africa, and calling for an end to their professions’ complicity with apartheid oppression. More recently, the medical professions have emphasized the ethical responsibility for all health-care professionals to speak out, and stand against any and all actions and injustices that create injuries and impairments (Horton, 2024; Shekhani & Jafarey, 2024). Even the conservative *New England Journal of Medicine* has broken its lengthy silence and published an article on the genocide (see Abu Fraiha et al., 2025).

### How Does This Relate to Occupational Therapists’ Everyday Practice?

It goes without saying that the practice of occupational therapists in Gaza has been profoundly affected by the current chapter in this genocide, to the point of risking their lives while providing services (Babish et al., 2024). Beyond Gaza, the forced displacement provoked by this war and the exponential production of disability, means that occupational therapists working with Palestinians who have fled their homes are also impacted (Fabianek et al., 2023). But this global conflict also affects the everyday practices of occupational therapists working in Canada. The following vignettes are de-identified scenarios illustrating what we are witnessing. They reveal the interconnected nature of experiences across the globe. In sharing these we consider the words of Irish poet, conflict mediator, and peacemaker, Padraig Ó Tuama (2022): “a single experience can sometimes contain worlds of meaning” (p. 69).

Eiman, a Palestinian Canadian, has been seeing an occupational therapist for several months. Her academic functioning is impacted by cognitive challenges and stark variations in energy due to comorbid traumatic brain injury and long COVID. She is currently on academic leave following the murder of family members in Gaza and is reflecting on her feelings about planning her return to university. Eiman says: *“Living here in Canada, we are on someone else's Palestine”;* linking Canada's treatment of Indigenous People and their land with Israel's treatment of Palestinians. The occupational therapist remains wordless in the face of Eiman's goal-setting challenge, not knowing how to proceed.

A group of occupational therapy students approach their instructor to discuss their final project based on a population of their choice. They have read up on the shifting immigration and refugee policies in Canada and want to work with incoming refugees from Gaza. They ask, in a furtive whisper, whether their choice of population “*will offend anyone and be considered antisemitic?”*

Sam is a Jewish man who sees an occupational therapist for occupational challenges related to an autoimmune disorder and chronic pain. His grandparents survived the Nazi Holocaust and came to Canada as refugees. Sam has been participating in peaceful anti-genocide protests this past year. The physical toll has significantly impacted his pain levels. He asks his occupational therapist to propose a new routine that prioritizes his political activities which include the possibility of arrest. The occupational therapist wonders about the risk to themselves if they chart this as part of their intervention – are they allowed to support activities possibly deemed “illegal”? What if this is seen as offensive to the political beliefs of the other interprofessional team members working with Sam?

Occupational therapy students who want to participate in student encampments and protests supporting Palestinians ask whether being arrested for “mischief” would affect their eligibility for professional licensure once they graduate. Their educators and regulators provide no clear response.

These vignettes illustrate how global conflicts affect the practice of occupational therapists in Canada. In October 1940, just before the Nazi Holocaust was officially recognized, the Honorable George Hoadley wrote a commentary in *CJOT* urging our profession to “weigh in the balance.” He wrote: “We are at war. To what degree are we competent from the standpoint of health, to do our part as a nation in this world struggle?” (p. 64). And here we are again. For therapists, it is crucial to receive guidance and support in navigating difficult conversations as they arise in clinical and educational settings, while also demonstrating some level of competence in addressing challenging conversations “on social injustices and inequitable opportunities for occupation” (ACOTRO et al., 2021, p. 14). Hoadley further emphasized: “we want to make sure we are not refusing to do our part in thinking through this problem. … Leadership is the awareness of need plus vision. No leadership is possible without disgust with the present situation” (1940, p. 72). He concluded with a call to action: “the power for better things *is within each one of us*, when our social conscience is aroused” (ACOTRO et al., 2021). By engaging with our “social conscience,” and in alignment with our professional obligations, these vignettes underscore the potential harm caused by adopting a “neutral” or silent stance in the face of such conflicts, and the need for leadership.

### What Can *We* Do as Individual Therapists, and as a Profession?

Though some, perhaps many, have already been acting, other students and therapists have been asking “What can we do?” Faced with the relentless horrors of genocide—unfolding before us in real time day by day, hour by hour, minute by minute—this pressing question can feel overwhelming. Our aim here is to break down the process of taking actions, both as individual therapists and collectively as a profession, so that we feel equipped, hopeful, and driven to act. We will focus on: (a) commitment to learning, (b) reflexive and reflective personal work, (c) the use of guiding frameworks, (d) building community, and (e) compassionate dialogue and action. Throughout this section, links are made, in the footnotes, to relevant Canadian Competencies (ACOTRO et al., 2021), and reflective prompts are provided, to guide occupational therapists who want to engage more in this process.

#### Commitment to learning

The Palestine–Israel violence did not start on October 7, 2023 (Pappe, 2024). While we do not need to know a detailed history of everything that has happened to acknowledge our feelings of horror and grief, we can and must commit to learning.^3^ We can read, watch, listen and learn, especially from the voices of those currently in—and those who have fled from—occupation and genocide. We can think deeply, critically, about what we learn.^4^ Doing so alongside others is especially helpful. We can question whose histories have been erased or overwritten, which voices and what stories are missing or marginalized in western news, history books, and formal education. We can pay attention to the language used to describe people and how this influences for whom we feel grief, empathy, and solidarity; what voice is used to describe war crimes and genocide (i.e., active or passive) and how this shapes accountability. We can search for the broader historical and political factors that silence some voices and amplify others. We can refuse the discursive attempts to reduce the humanity of any group of people and ground ourselves in compassion and solidarity. We can share this learning within our spheres of influence.

Histories of oppressions and injustices matter.^5^ They can teach us how systems have worked historically to reinforce and reward those who remain silent and inert, while demonizing those who have challenged power in favor of peace and justice. They help us name the injustice against which we seek to take action. Paradoxically, and to employ an “occupational” lens: the labor associated with peacebuilding has been largely unpaid, perhaps because historically it was extensively done by women (Osman Ibnouf, 2020). Like other historical injustices, this labor has been subject to massive state-sanctioned repression and criminalization (Duhart, 2019). It is therefore not surprising that, with few exceptions, most peacebuilding efforts globally, including diplomatic efforts, but also protests, writing letters, signing petitions, and other forms of activism, have been done outside official roles and positions, due to worry about and threats of losing employment and professional affiliations. How can we not be concerned when peacebuilding efforts can so easily be transformed into criminal offenses? In our professional contexts, these efforts are often labeled as “unprofessional” (Beagan et al., 2024), even though they embody the very principles of equity and justice that our professional competencies, oaths, and laws/policies demand in the face of occupational injustices and human rights violations.

#### Reflexive and reflective personal work

Reflexive and reflective personal work needs to take place alongside ongoing learning to examine our own embodied emotions, biases, triggers and defenses, and our own lived experience with this and other forms of injustice.^6^ While such individual reflection can be challenging and appear far removed from collective action, this work helps us take intentional actions aligned with justice values even when under pressure, rather than reverting to habitually learned responses, such as remaining silent (Haines, 2019) and self-censoring. Here, we can draw from resources, both from within occupational therapy (e.g., Lerner & Kim, 2022; Sterman & Njelesani, 2021; Turcotte & Barlott, 2024; Zafran, 2021, 2024) and beyond (e.g., Menakem, 2017), that can help challenge our internalized supremacist beliefs.

In this work, we can ask ourselves: what do I notice happening within my body? Where in my body do I notice responses? Are these responses familiar? Can I tell the difference between feelings of discomfort or threat and actually being unsafe? What emotions do these sensations convey? What internal resources do I have that can help me stay with these emotions without getting overwhelmed or shutting down? What thoughts or sense-making related to these emotions arise in me? When? What kind of action/movement/inaction/inertia do these thoughts trigger for me? With deeper self-awareness, we can act while respecting our own needs and those of others.

Engaging in deep reflection on core values is essential to ground this work in a meaningful way. Understanding our values (both personal and professional) and how they do or do not support solidarity with, and justice for, those who are oppressed in a given situation can further our critical questions, improve our tolerance for exploring opposing narratives, identify moments of resistance, minimize obstacles to others’ efforts towards justice, and allow us to be responsive to the rights and agency of those with whom we stand in solidarity without infringing on the fundamental (vs. ideological) rights of others. This reflexive process shapes the strategies we employ and ensures that our actions are both intentional and consistent with our larger ethical and professional obligations.

#### Guiding frameworks

Using a guiding framework that describes different roles people and communities can take in anti-oppression work may be helpful. One framework is Deepa Iyer's Social Change Ecosystem Map (Building Movement Project, n.d.). Centered on collective values of equity, liberation, justice, and solidarity, the Social Change Ecosystem Map describes 10 roles through which we (as individuals or as a profession) can take action, including disruptors, guides, and frontline responders. Another framework, by the collective Slow Factory (n.d.) describes 20 roles, including advocate, researcher, and communicator. Such frameworks complement our understanding of occupational participation in political action/spheres, illuminating the diverse roles we can assume in challenging oppressions.^7^ As human beings, we bring a vast array of lived experiences, gifts, capacities, strengths, and values to this work. These frameworks also anchor action in community, reminding us that anti-oppressive action—such as action against the genocide of Palestinians—always requires collective action.

Moreover, these roles are not fixed and nor are we; we can navigate between them, rather than being confined to a single approach. We can be on-the-streets activists, engaging in protests; we can be communicators, writing, reading, and sharing stories and poetry to bring awareness of the horrors of the genocide; we can be problem solvers by reaching out to a colleague who has been directly impacted or by organizing and/or contributing to mutual aid efforts. We also acknowledge that the risks of openly opposing injustice differ for each of us, depending on our social identities and places in power hierarchies. It is essential that we critically appraise risks with both honesty and self-compassion, carefully distinguishing between the silence of presumed “neutrality,” the silence of minimizing personal or community harm for those already in marginalized or precarious situations, and/or the justification of silence due to an unwillingness to risk anything in the advancement of justice (Wyatt et al., 2024).

#### Finding and fostering community

Collective action through community is safer, more fulfilling, effective, and sustainable when opposing long-standing injustice. We can be in community and act in solidarity by joining existing groups of occupational therapists or other health professionals who are acting against the genocide of Palestinians.^8^ We can quietly or openly signal to others our desire to have conversations with existing communities—such as the CAOT Justice, Equity, Diversity, and Inclusion practice network—or with others in our immediate contexts. Engagement in community can lead to collectives of like-minded and like-hearted people; people with whom we share those foundational values that drive this work.^9^

Even relatively simple actions, such as using the words “Palestine” and “genocide” instead of “conflict in the Middle East,” or distinguishing between “the state of Israel” and “Jewish people,” can signal openness to complex conversations and build connections. We can use images or symbols to display our engagement with reconciliation, transformative justice, and/or our opposition to genocide. We can raise questions and invite questions, such as providing opportunities to engage with the implications of the UN reports in curricula, research, and practice. We can express deep feelings about the violent oppression of Palestinian and Lebanese peoples *as well as* the losses experienced by Israelis and Jewish people. We can co-create connections through visible solidarity with those suffering, and in doing so, build and nurture communities to do this critical work.

We invite you to ask yourself: what communities do I already engage with? Have I witnessed signs within my current groups of shared concern about the genocide of Palestinians? How can I signal my desire to connect and build community? What existing communities resonate with my desire to act? How can I build connections with like-minded, like-hearted people to co-create a community of action?

#### A call for compassionate dialogue and actions in solidarity

Compassion is a necessary component for engaging in difficult conversations that are respectful and inclusive. Within occupational therapy education, students and educators need spaces that support reflection, reflexivity, humility, expressions of emotion, analysis, and honesty. Colleagues, managers, and other patrons need conversations that acknowledge their confusion, grief, (out)rage, pain, and fear; that strive for clarity regarding injustices; that hold ourselves and each other accountable to the espoused commitments of our profession. We can draw on the skills and strategies of trauma-informed practices to better understand our automatic, embodied responses, building capacity to engage in challenging and necessary dialogue (Jones, 2024). We can draw on nonviolent communication, centering empathy, honesty, and self-reflection to build connection and understanding that grounds further action (Rosenberg, 2015). We can invite experienced mediators in to help co-create such spaces (e.g., https://www.pledj.org/solidarity-dialogues). We need to speak at each strategic or possible opportunity to do so, not speaking *for* or “on behalf of” anyone, but speaking when we know in our hearts that something is terribly, terribly wrong.

Engaging in the anti-oppressive practices required by our national professional competencies means making space for service-users and peers to connect with us about how this genocide is affecting their occupations, their lives, their selves, and amplifying the voices and rights of those who are pressed into silence. It means bearing witness, understanding what is shared through the lens of systemic oppression and power-dynamics rather than pathology or demonization, and choosing approaches that center communal healing and resistive/collective occupations (Zafran, 2021, 2024). It means supporting those with whom we work—including service-users, colleagues, and students—to understand their legal and ethical rights, facilitating connections with each other, and linking them with community resources and activists. It means challenging ourselves and our professional leaders by asking questions that encourage us, as occupational therapists, to apply our professional competencies to real-world situations.^10^ We can support our professional leaders to learn from their actions and inactions, to model growth, and accountability, to transform policies and decision-making within complicit systems, and to keep trying … again and again.

We draw on diverse cultural, theoretical, and experiential resources in comprehending how to identify and narrate injustice (Zafran, 2025). We will not always agree with each other, even amongst those of us confronting the same injustices (AnonymOT Collective, 2025; Babish, 2025); even among those of us who authored this paper. But we can always work in solidarity. As Puerto Rican Jewish poet Aurora Levins Morales explains, “Solidarity is not a matter of altruism. Solidarity comes from the inability to tolerate the affront to our own integrity of passive or active collaboration in the oppression of others” (2019, p. 125). We must recognize the passion and determination in one another, as we resist different yet intersecting oppressions, and we must find ways to support our connected struggles.

Oppression is intended to divide us: to obscure our witnessing of and understanding of injustices and to hinder us from recognizing where our struggles may serve to unite us. Oppression isolates through disconnection, fear, reprisals, and violence. In that context it takes courage to speak up and to be open-hearted in our actions. But silence does not protect us from harm; indeed, it does the opposite. Reflecting on times she had “betrayed [herself] into small silences,” Black lesbian feminist poet Audre Lorde^11^ wrote:

I have come to believe over and over again that what is most important to me must be spoken, made verbal and shared, even at the risk of having it bruised and misunderstood… To question or to speak as I believed could have meant pain, or death. But we all hurt in so many ways, all the time… My silences had not protected me. Your silence will not protect you… We can learn to work and speak when we are afraid… while we wait in silence for that final luxury of fearlessness, the weight of that silence will choke us. (Lorde, 1984, pp. 40–44)

## Conclusion

A ceasefire in Gaza beginning January 19, 2025, was terminated by Israel on March 18, 2025 killing an estimated 400 people in one day (The Guardian, 2025). The onslaught is ongoing. All critical infrastructure has been destroyed, including health care, education, transportation, sanitation, and food supplies, with Gaza's soil and groundwater contaminated by munitions and toxins, and the air polluted by burning buildings (Ahmed et al., 2024; Sah & Dawas, 2024). Beyond the “exceptionally high” number of civilian deaths directly caused by bombs and guns (Jamaluddine et al., 2025), a “slow-motion genocide” continues (Docker, 2012; Lendman, 2010; Nijim, 2023; Pappe, 2024; Robinson, 2024; Third World Approaches to International Law Review, 2023), due to mines left under the rubble, starvation and malnutrition, lack of water and shelter, poor air quality, exposure to the elements, and loss of agriculture.

The illegal annexation of and violence in the West Bank is also increasing (Landay & Pamuk, 2025), while Israel's unlawful occupation has further extended into southern Lebanon and Syria. The toll of multigenerational violence cannot be fathomed. However, one should not underestimate the capacity of Palestinians—on their lands and in the diaspora—to endure, resist, return, and rebuild. This genocide “is about the social fabric, how we view ourselves, how we view others. … Only acts of international solidarity, and justice, and redress can contribute to this healing process” (Jabr quoted in Bedaiwy, 2024). There is a long journey ahead of us, as occupational therapists, not just to support rebuilding structures and capacity, but also to earn (back) trust from affected communities.

Some occupational therapists have been speaking out (Babish et al., 2024; Palestinian Association of Occupational Therapists, 2024; Zafran, 2024, 2025), taking actions within the profession and beyond, working through the shame of witnessing the silence of our profession and resisting complicity (AnonymOT Collective, 2024; Turcotte & Barlott, 2024). Some occupational therapists are making the connections between genocide of Palestinians and other contexts of violent occupation; interconnected issues which our professional leaders urgently need to engage with. Our professional organizations and institutions are failing to demonstrate our professional competencies and espoused commitments, and are even blocking therapists and students from enacting these professional obligations.

On October 25, 2023, 3 weeks into the attacks on Gaza, Egyptian‒Canadian novelist and journalist Omar El Akkad (2025) posted a tweet predicting that “One day, when it's safe, when there's no personal downside to calling a thing what it is, when it's too late to hold anyone accountable, everyone will have always been against this.” As a profession of morally engaged individuals, we need to come together to do better, resist silence, welcome dialogue, enact our professional and ethical commitments (Hammell et al., 2022), and support occupational justice, self-determination, and human rights locally and globally.

## Key Messages

- The occupational therapy profession has failed to act in accordance with its own statements concerning the obligation to uphold occupational and human rights. Silence and silencing—which have harmed members of our professional community—support the *status quo* of injustice and do not constitute “neutrality.”
- Occupational therapy professional organizations must align their actions with professional competencies and commitments that demand an anti-oppressive, equity-focused stance, and with their obligation to advance human rights and social justice.

## Notes

1. Over 4000 Lebanese have been killed and 1.2 million displaced, including ongoing deaths in contravention of a ceasefire (ref: https://www.bbc.com/news/articles/czeprnw4repo).

2. Not to exclude groups who are disabled, LGBTQIA+, Afro-Palestinians, and so on.

3. D3.1. Stay aware of political, social, economic, environmental, and technological effects on occupational therapy practice.

4. C1.3. Challenge biases and social structures that privilege or marginalize people and communities.

5. C1.2. Analyse the effects of systemic and historical factors on people, groups, and their occupational possibilities.

6. C2.2. Practise self-awareness to minimize personal bias and inequitable behavior based on social position and power.

7. B1.4. Adjust to power imbalances that affect relationships and communication. C3.6. Advocate for environments and policies that support sustainable occupational participation. C1.3. Challenge biases and social structures that privilege or marginalize people and communities.

8. Disrupt OT, Occupational Therapists for Palestine, Jewish OTs for Palestine, Irish OTs for Palestine, AnonymOT Collective, Health Workers for Palestine.

9. C2.1. Contribute to a practice environment that is culturally safer, anti-racist, anti-ableist, and inclusive.

10. E1.7. Be accountable for all decisions and actions made in the course of practice. F2.5. Act responsibly when there are environmental or social impacts to their own behavior or advice, or that of the team. F4.2. Advocate for an alignment between occupational therapy standards and processes, organizational policies, social justice, and emerging best practices.

11. We honor the longstanding solidarity of the Black Liberation movement in the United States of America with the Palestinian Liberation movement. Key actors include Assata Shakur, Angela Davis, Audre Lorde, and more recently, Ta-Nehisi Coates. In the words of Black poet June Jordan in her poem “Moving Towards Home,” “I was born a Black woman/and now/I am become a Palestinian…” (Jordan, 2021, p. 93).
